# Cystathionine- γ-lyase promotes process of breast cancer in association with STAT3 signaling pathway

**DOI:** 10.18632/oncotarget.20057

**Published:** 2017-08-07

**Authors:** Jing You, Xiaoyan Shi, Huimin Liang, Juan Ye, Lupeng Wang, Huanxiao Han, Hongyu Fang, Wenyi Kang, Tianxiao Wang

**Affiliations:** ^1^ College of Pharmacy, Henan University, Kaifeng 475004, Henan Province, China; ^2^ Huaihe Hospital, Henan University, Kaifeng 475000, Henan Province, China

**Keywords:** cystathionine-γ-lyase, H2S, transcription factor STAT3, breast cancer, growth

## Abstract

Here we provide evidences to link cystathionine-γ-lyase (CSE) to the development of breast cancer. CSE expression is up-regulated in both breast cancers and breast cancer cell lines and results in proliferation and migration of breast cancer cells. CSE Function in breast cancer depends on the STAT3 signaling pathway, a regulator of critical cell functions including cell growth in a wide variety of human cancer cells via activating the expression of relative genes. STAT3 positively relates to CSE expression. It activates the CSE promoter via a direct binding to the promoter. Moreover, CSE could reversely regulate STAT3 expression and consequently enhance the effect of STAT3 on CSE. Taken together, these data demonstrate for the first time the roles of CSE in breast cancer leading to breast cancer development in association with STAT3 signaling pathway.

## INTRODUCTION

Breast cancer is the most frequent cancer worldwide in women and the sixth leading cause of female cancer death in China. Breast cancer is classified into different subtypes mainly based on the status of biomarkers ER/PR and Her2, which lead to different treatment and prognosis [1]. However, identification of new biomarkers and new genes involved in cancer progress may provide novel approaches for diagnostic and prognostic evaluation.

Hydrogen sulfide (H_2_S) has been concerned mainly as toxic gas and an environmental pollutant for many decades. In 1990s, endogenous H_2_S was found to exist in various tissues and organs of the organism. As the third gasotransmitter signaling molecule alongside nitric oxide (NO) and carbon monoxide (CO) [2–7], it plays important roles in many physiological processes. Endogenous H_2_S is mainly generated by two pyridoxal-5-phosphate (PLP)-dependent enzymes, cystathionine-γ-lyase (CSE) and cystathionine-β-synthase (CBS) [2]. Early studies revealed that CSE is prevalently expressed in many tissues except central nervous system [2], while recent ones demonstrated that endogenous H_2_S produced by CSE promotes proliferation of human hepatoma and colon cells [8–9]. However, in breast cancer, the bio-functions of CSE/H_2_S system have not been understood yet. This promotes our investigation into the roles of CSE in breast cancer development and progression.

One approach to illustrate the biological functions of CSE gene is to explore its upstream signaling molecules. Previous studies showed that PI3K/Akt pathway can regulate CSE gene expression via transcription factor specificity protein 1 (Sp1) to promote hepatoma cell growth [8]. Wnt pathway can also induce the transcription of CSE gene expression by β-catenin to facilitate colon cancer cell proliferation [10]. Signal transducer and activator of transcription 3 (STAT3), a transcription factor that regulates critical cell functions, is constitutively activated in a wide variety of human cancer cells and plays significant roles in cancer cell growth by regulating the expression of relative genes[11–15]. Currently, the upstream signaling molecules of regulating CSE gene expression in breast cancer are poorly understood. Here we found that CSE protein level is positively correlated with STAT3 protein expression in breast cancer, implying the involvement of STAT3 in upstream regulation of CSE expression. Both loss-of-function and gain-of-function studies indicated that CSE functions as a potential tumor promoter. Further, transcription factor STAT3 directly targets CSE, which mediates CSE function as a tumor activator.

## RESULTS

### CSE expression is up-regulated in breast cancer

**Figure 1:**
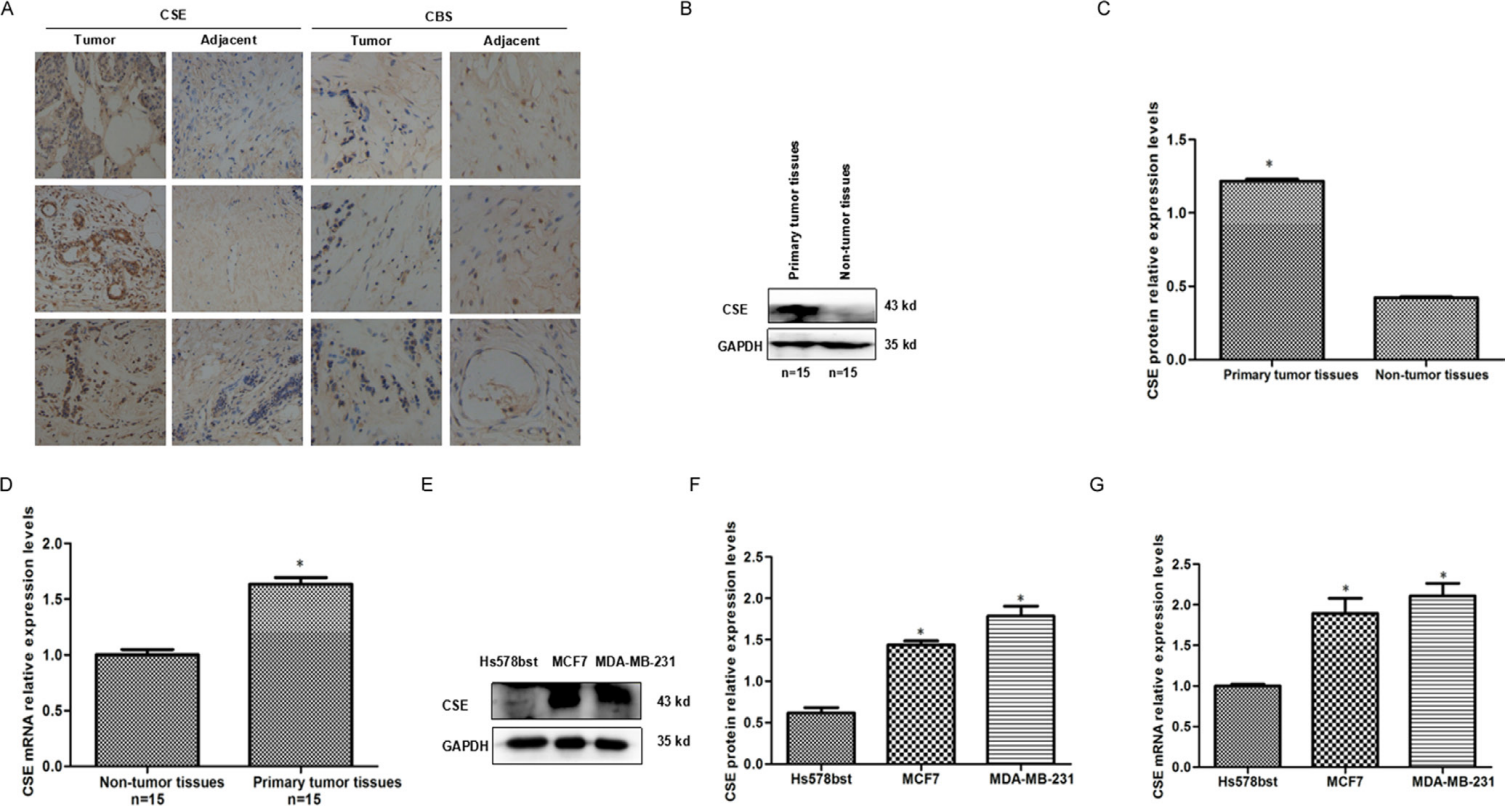
Expression patterns of CSE in breast cancer tissues and breast cancer cell lines (**A**) Immunohistochemistry (IHC) analysis of CSE and CBS expression levels in breast cancer tissues and adjacent samples. CSE is highly expressed and higher than CBS level in breast cancer tissues. Immunohistological staining assays were performed with an anti-CSE antibody (diaminobenzidine (DAB) staining, scale bars, 100 μm). (**B** and **C**) WB and quantitative analysis of CSE protein levels in breast cancer tissues and adjacent samples. 15 tumor tissues and 15 adjacent non-tumor tissues were analyzed by WB. CSE expression is up-regulated in breast cancer tissues compared with non-tumor tissues. GAPDH was used as an internal control. *P < 0.05 vs non-tumor tissues. (**D**) qRT-PCR analysis of CSE mRNA levels in breast cancer tissues and adjacent samples.15 tumor tissues and 15 adjacent non-tumor tissues were analyzed. CSE mRNA level is significantly up-regulated in breast cancer tissues. *P < 0.05 vs non-tumor tissues. (**E** and **F**) WB and quantitative analysis of CSE protein levels in breast cancer cells and mammary epithelial Hs578bst cells. *P < 0.05 vs mammary epithelial Hs578bst cells. (**G**) qRT-PCR analysis of CSE mRNA levels in breast cancer cells and mammary epithelial Hs578bst cells. Error bars indicate s.d. (*n* = 3). *P < 0.05 vs mammary epithelial Hs578bst cells.

To explore the expression patterns of CSE in breast cancer tissues, we compared primary tumor with non-tumor tissues by quantitative RT–PCR (qRT–PCR), western blot (WB) and immunohistochemistry. The results showed that CSE expression was significantly up-regulated in breast tumors compared with the adjacent non-tumor tissues (Figure 1A–1D). In addition, we also observed the increased mRNA and protein levels of CSE in breast cancer MCF7 and MDA-MB-231 cell lines when compared with mammary epithelial cell line Hs578bst (Figure 1E–1G). The results suggested that CSE expression is up-regulated in breast cancer.

### Knockdown of CSE inhibits proliferation and migration

**Figure 2:**
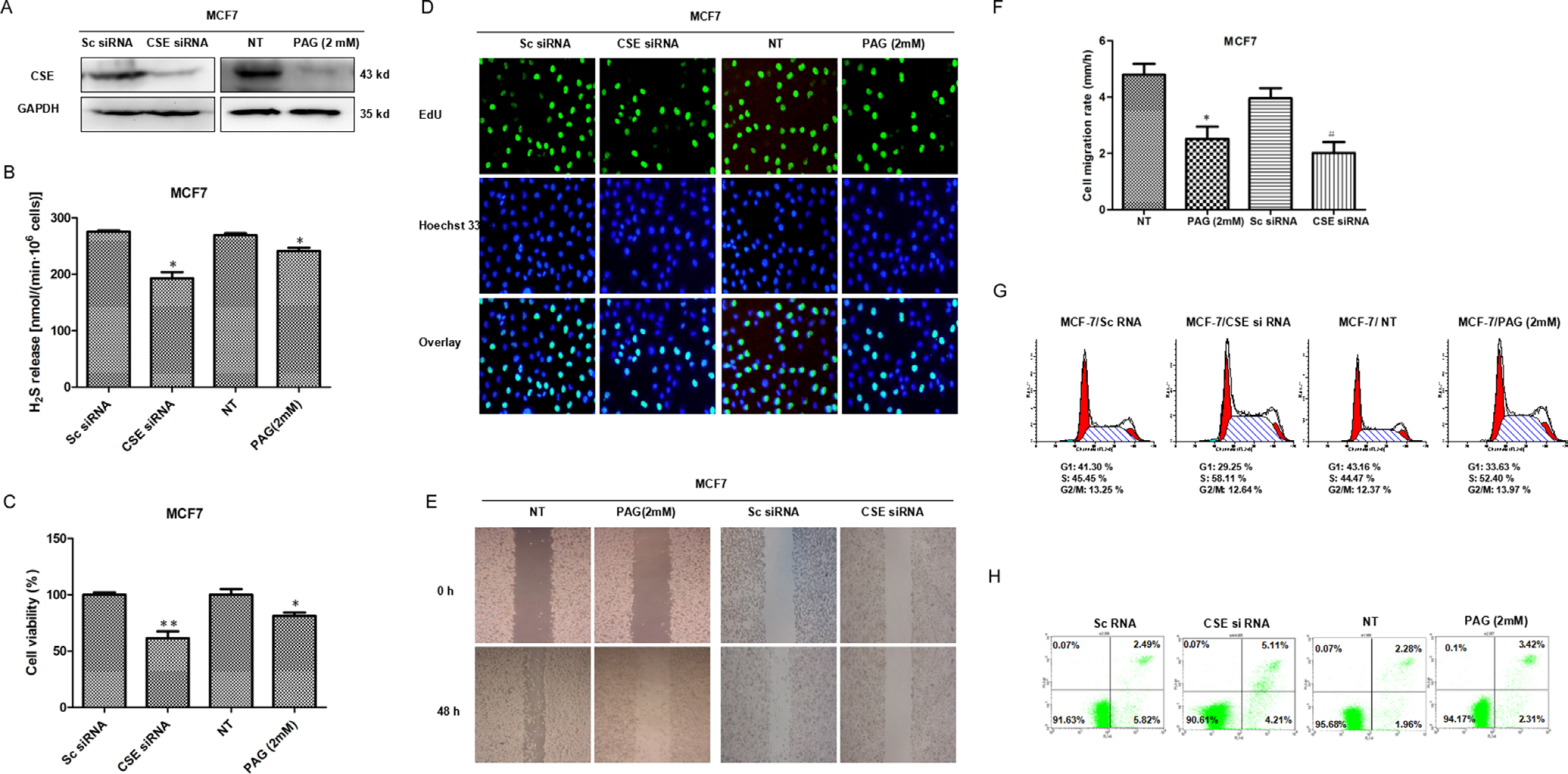
Effects of CSE knockdown by siRNA or inhibitor on cell proliferation, migration and apoptosis in MCF7 cells (**A**) The knockdown expression of CSE was confirmed by WB in MCF7 cells. GAPDH was used as an internal control. (**B**) Methylene blue assay was used to examine the effect of CSE knockdown on H2S production in MCF7 cells. *P < 0.05. (**C**) MTS assay was used to detect the effect of CSE knockdown on proliferation in MCF7 cells. *P < 0.05; **P < 0.01. (**D**) The effect of CSE knockdown on cell proliferation was further confirmed by EdU assay. EdU, 5-ethynyl-2’-deoxyuridine. (**E** and **F**) Scratch assay was performed to evaluate the effect of CSE knockdown on cell migration. *P < 0.05 vs NT treatment group. ^#^*P* < 0.05 vs Sc siRNA group. (**G**) Cell cycle was analyzed by flow cytometry. (**H**) Cell apoptosis was detected by Annexin V/FITC double staining.

To explore the potential role of CSE in breast cancer, we firstly knocked down CSE with siRNA or inactivated CSE with inhibitor in MCF7 cells. WB and Methylene blue assay showed that both CSE expression and H_2_S production were significantly reduced in the MCF7 cells transfected with siRNA or treated with inhibitor PAG (Figure 2A and 2B). We then detected the effects of knockdown CSE on cell proliferation. The MTS results showed that knockdown of CSE inhibited proliferation of MCF7 cells (Figure 2C). The inhibitory effect of CSE knockdown on cell proliferation was confirmed by EdU assays. CSE knockdown increased the number of EdU^+^ cells in MCF7 cell lines (Figure 2D). Meanwhile, the scratch assay was performed to evaluate the effect of CSE knockdown on cell migration. As shown in Figure 2E and 2F, CSE knockdown inhibited the migration of MCF7 cells. We also measured cell cycle and the percentage of apoptotic cells by flow cytometry analysis. The CSE *kd* MCF7 cells were found to be arrested in S phase (Figure 2G), but had no a significant higher percentage of apoptotic cells as compared with controls (Figure 2H). Taken together, these data demonstrated that CSE knockdown inhibited proliferation and migration in breast cancer cells.

### CSE overexpression promotes proliferation and migration

**Figure 3:**
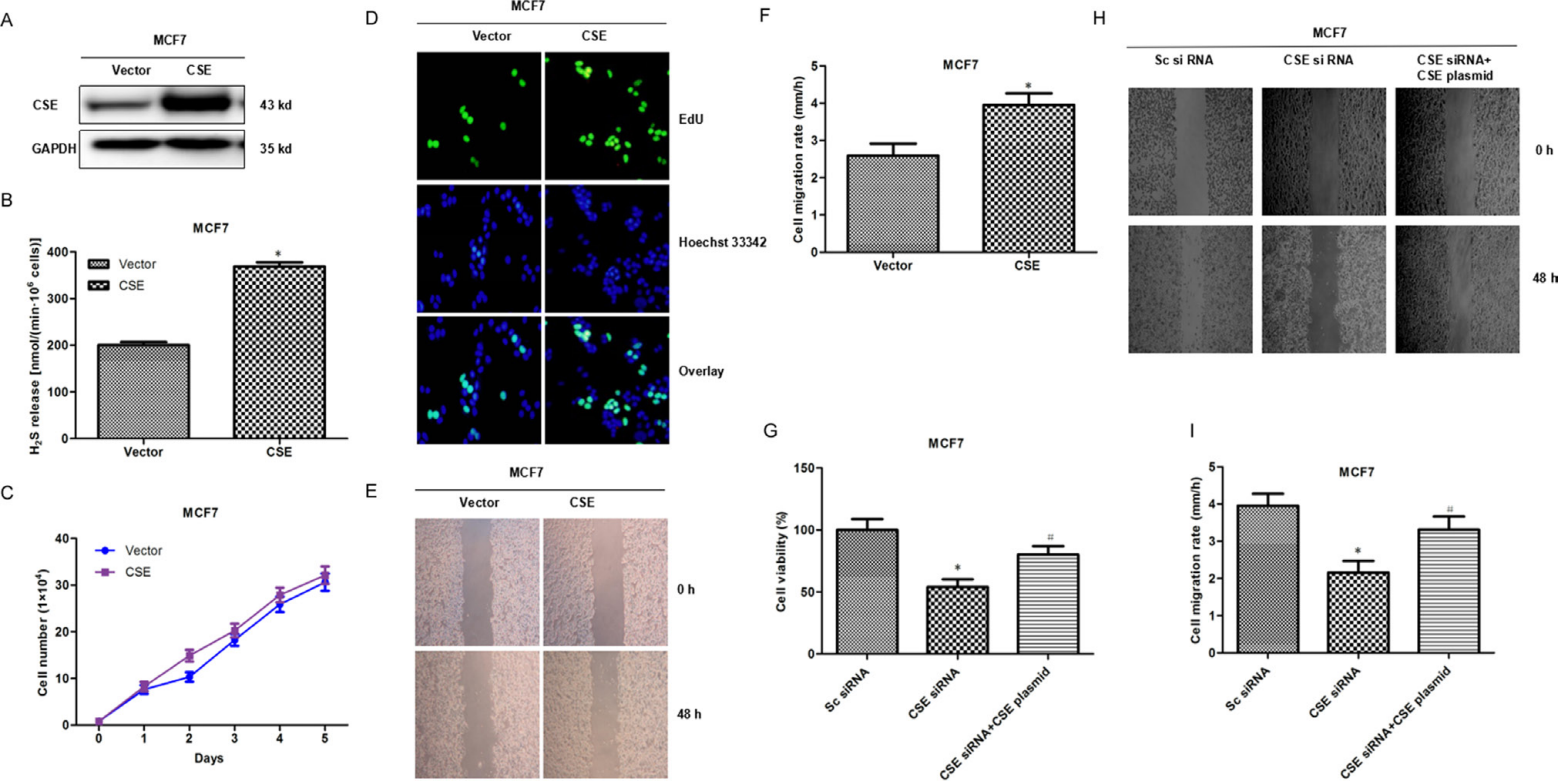
Analyses of cell proliferation and migration associated CSE overexpression in MCF7 cells (**A**) Transfectants of CSE and vector control in MCF7 cells were identified by WB. More abundant CSE was detected after CSE transfection compared with the control vector transfection. (**B**) Methylene blue assay was used to examine the effect of CSE overexpression on H_2_S production in MCF7 cells. *P < 0.05; (**C**) MTS assay was used to detect the effect of CSE overexpression on proliferation in MCF7 cells. (**D**) The effect of CSE overexpression on cell proliferation was further confirmed by EdU assay. EdU, 5-ethynyl-2’-deoxyuridine. (**E** and **F**) Scratch assay was performed to evaluate the effect of CSE overexpression on cell migration. *P < 0.05. (**G**) MTS assay was used to detect the effect of the co-transfection of CSE siRNA and CSE overexpressed plasmid on proliferation in MCF7 cells. *P < 0.05 compared with Sc siRNA group; ^#^*P* < 0.05 compared with CSE siRNA group. (**H** and **I**) Scratch assay was used to detect the effect of the co-transfection of CSE siRNA and CSE overexpressed plasmid on MCF7 cell migration. *P < 0.05 compared with Sc siRNA group; ^#^*P* < 0.05 compared with CSE siRNA group.

To further confirm the potential roles of CSE in breast cancer, we constructed further gain-of-function cell models by transfecting a CSE-expressing plasmid into human breast cancer MCF7 cells. The expression of exogenous CSE and level of H_2_S were confirmed by WB and Methylene blue assay (Figure 3A and 3B). The MTS assay, EdU assay and scratch assay analysis showed that CSE overexpression promoted cell proliferation and migration (Figure 3C–3F), compared with the negative controls. Meanwhile, we observed that the co-transfection of CSE siRNA and CSE overexpressed plasmid rescued the effects of cell growth and migration caused by CSE knockdown (Figure 3G–3I). These data together with the CSE knockdown results suggested that CSE might function as a potential tumor promoter.

### Transcription factor STAT3 promotes proliferation and migration in breast cancer cells

**Figure 4:**
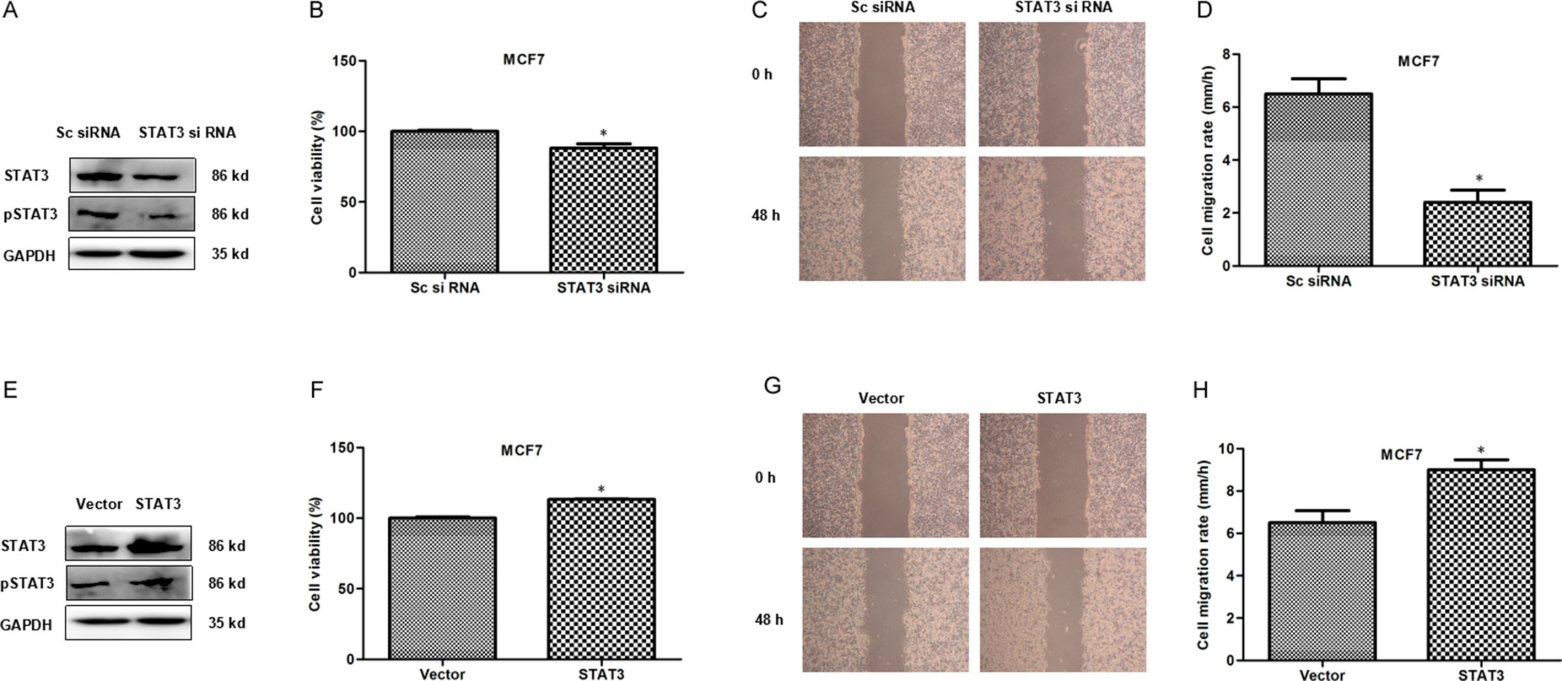
STAT3 promotes proliferation and migration in breast cancer cells (**A**) The knockdown expression of STAT3 and pSTAT3 was confirmed by WB in MCF7 cells. GAPDH was used as an internal control. (**B**) MTS assay was used to detect the effect of STAT3 knockdown on proliferation in MCF7 cells. *P < 0.05. (**C** and **D**) Scratch assay was performed to evaluate the effect of STAT3 knockdown on cell migration. *P < 0.05. (**E**) Transfectants of STAT3 and vector control in MCF7 cells were identified by WB. More abundant STAT3 was detected after STAT3 transfection compared with the control vector transfection. (**F**) MTS assay was used to detect the effect of STAT3 overexpression on proliferation in MCF7 cells.*P < 0.05. (**G** and **H**) Scratch assay was performed to evaluate the effect of STAT3 overexpression on cell migration. *P < 0.05.

STAT3, as a transcription factor, is highly activated in breast cancer cells and promotes cancer cell growth [11]. In this study we also observed that STAT3 knockdown inhibited proliferation and migration of MCF7 cells (Figure 4A–4D) while its over-expression promoted proliferation and migration (Figure 4E–4H). The results suggested that transcription factor STAT3 promotes proliferation and migration in breast cancer cells. Next we explore if CSE expression correlates with STAT3.

### STAT3 expression positively relates to CSE expression

**Figure 5:**
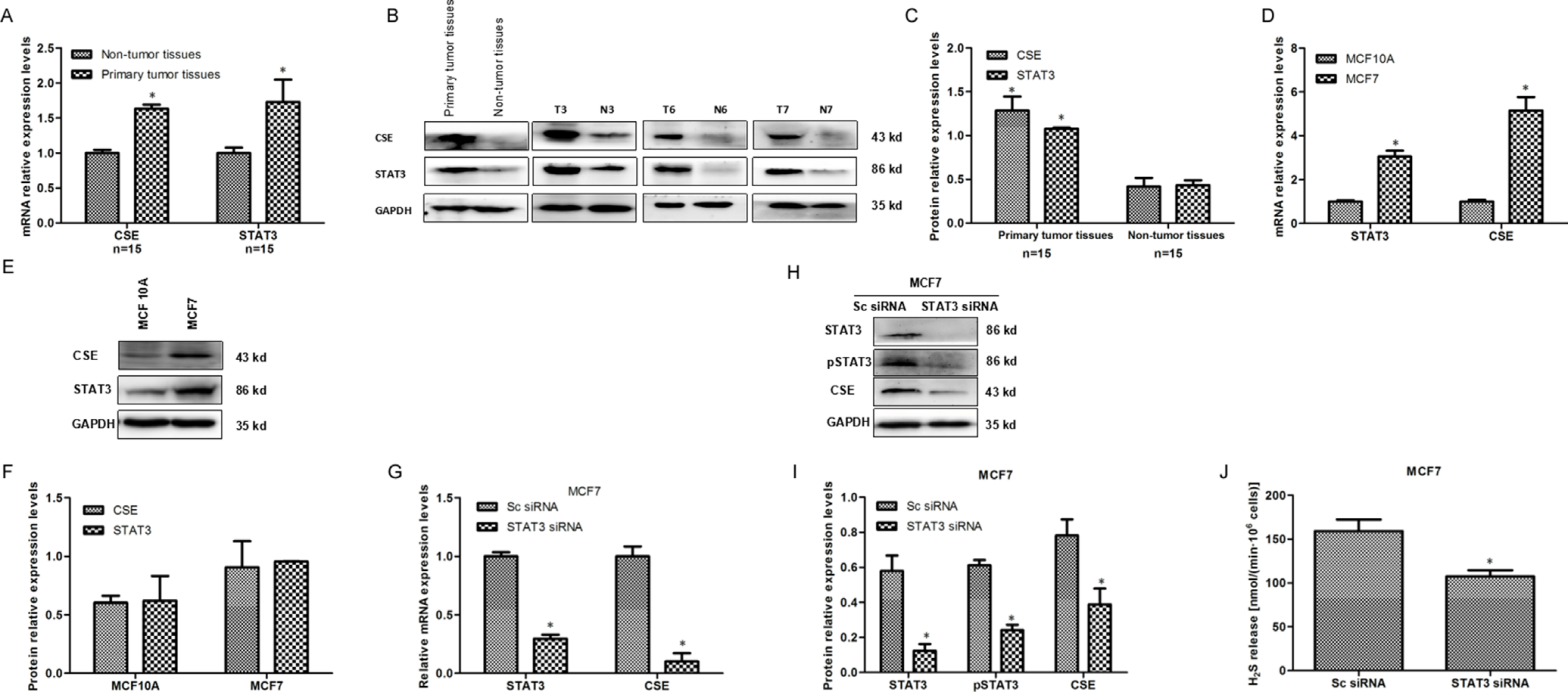
STAT3 positively relates to CSE expression in human breast cancer tissues and cells (**A**) qRT-PCR analysis of CSE and STAT3 mRNA levels in breast cancer tissues and adjacent non-tumor tissues. *P < 0.05 vs non-tumor tissues. (**B** and **C**) WB detection and quantitative analysis of CSE and STAT3 protein levels in breast cancer tissues and adjacent non-tumor tissues.*P < 0.05 vs non-tumor tissues. (**D**) qRT-PCR analysis of CSE and STAT3 mRNA levels in MCF7 cells and mammary epithelial MCF 10A cells. *P < 0.05 vs MCF10A cells. (**E** and **F**) WB and quantitative analysis of CSE and STAT3 protein levels in MCF7 cells and mammary epithelial MCF 10A cells. (**G**–**J**) Effect of STAT3 knockdown by RNAi on CSE mRNA, protein and H_2_S level in MCF7 cell line by qRT-PCR, WB and methylene blue assays. Error bars indicate s.d. (*n* = 3). *P < 0.05.

To explore the potential upstream regulators for CSE, we firstly investigated the correlation between STAT3 and CSE expression in human breast cancer tissues and cells. qRT-PCR and WB results showed that both mRNA and protein levels of STAT3 were up-regulated in CSE-overexpressed human breast cancer tissues (Figure 5A–5C) and human breast cancer cell line (Figure 5D–5F), which suggested that STAT3 is positively related to CSE expression. To further determine the contribution of STAT3 in CSE expression, the expression of CSE in MCF7 cells transfected by STAT3 siRNA was examined by qRT–PCR and WB. The results indicated that CSE was decreased markedly both at mRNA and protein levels in MCF7 cells when STAT3 was knockdown (Figure 5G–5I). H_2_S level was also significantly decreased in MCF7 cells transfected by STAT3 siRNA (Figure 5J). Taken together, these data suggested that CSE was the possible target gene of STAT3 in breast cancer.

### STAT3 directly targets CSE

**Figure 6:**
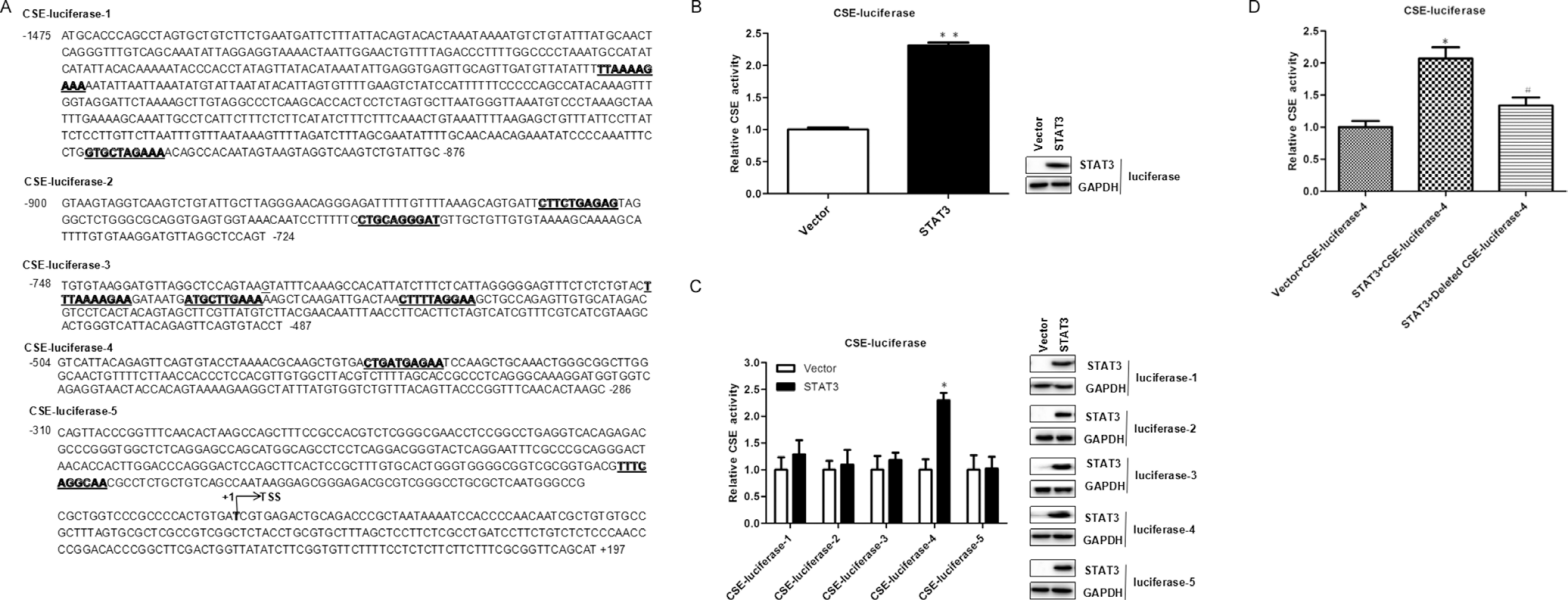
STAT3 directly binds to promoter of CSE (**A**) The five different regions of the CSE promoter were constructed into luciferase reporter. The possible binding sites of STAT3 in CSE promoter region are underlined with bold font; TSS, transcription start site. (**B**) STAT3 directly targets CSE. A luciferase reporter linked with the full-length native promoter of CSE was used for the luciferase reporter assay in 293T cells. Results were normalized with internal controls and presented as averages with SD from three experiments. (**C**) Different partial regions of CSE promoter were analyzed by luciferase reporter assay. Luciferase reporters linked with partial native promoter regions of CSE were used for the luciferase reporter assay in 293T cells. WB analysis of STAT3 expression was performed to exclude that the differences in transcriptional activity reflect changes in expression. The STAT3-binding region in CSE promoter should be at −504 to −286 according to the luciferase reporter assay results of the five different CSE-luciferase reporters. According to the binding sites predicted by Jaspar, the direct binding sites is likely located at CTGATGAGAA (−464 to −454) of the CSE promoter region. (**D**) The effect of STAT3 on human wild-type and deleted CSE promoter activity in 293T cells. CTGATGAGAA is deleted in CSE-luciferase-4. Error bars indicate s.d. (*n* = 3). *P < 0.05 compared with Sc siRNA group; ^#^*P* < 0.05 compared with CSE siRNA group.

To investigate whether CSE is a direct target of STAT3, we searched the STAT3 transcription factor-binding sites in CSE promoter using Jaspar (http://jaspar.genereg.net/). Several STAT3 transcription factor-binding sites were identified in CSE promoter region (Figure 6A). We speculated that STAT3 might regulate CSE transcription by directly binding to its promoter region. To verify this hypothesis, we determined the promoter activity of CSE gene. Firstly, the full CSE promoter was amplified and inserted into the pGL3-Basic vector and then the CSE promoter- pGL3-Basic recombinant plasmid and STAT3-wt plasmid were transiently co-transfected into the 293T cells. The luciferase assay results showed that overexpression of STAT3 significantly enhanced the activity of CSE promoter (Figure 6B).

To examine the STAT3-binding sites in the CSE promoter, five different regions (−1475 to −876, −900 to −724,−748 to −487, −504 to −286, −310 to +197) of the CSE promoter were analyzed by luciferase reporter assays (Figure 6C) and the STAT3-binding site was very likely located at the CTGATGAGAA (−464 to −454) of the CSE promoter region (Figure 6C) using Jaspar (http://jaspar.genereg.net/) searching. These findings demonstrated that CSE was a direct target gene of STAT3. To further investigate whether STAT3 activates the CSE promoter through association to the binding site (CTGATGAGAA), we deleted the site (CTGATGAGAA) in the CSE promoter, which caused the elimination of the stimulating effect (Figure 6D). The results indicated that CSE is a direct target of STAT3.

### CSE reversely acts on STAT3

**Figure 7:**
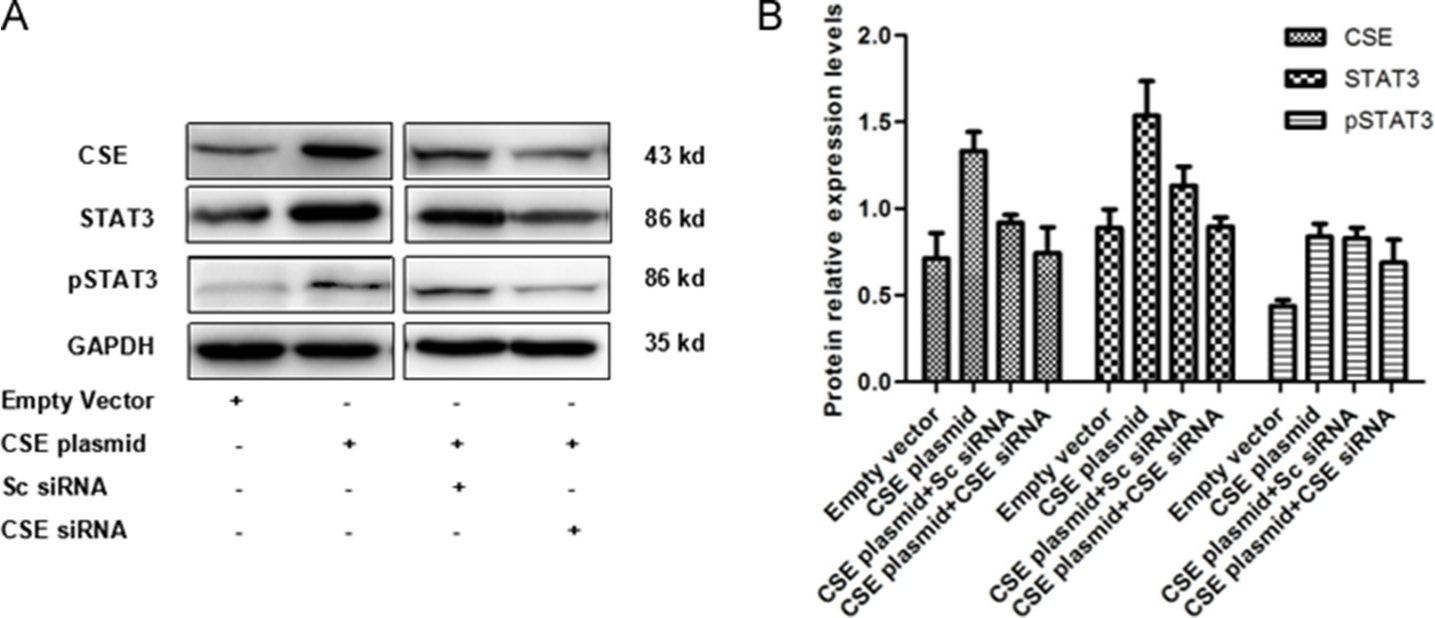
Effect of CSE overexpression and knockdown on STAT3 protein level in MCF7 cells (**A**) WB analysis of STAT3 and pSTAT3 protein levels in CSE overexpressed and /or down-regulated MCF7 cells. (**B**) Quantitative analysis of STAT3 and pSTAT3 protein levels in CSE overexpressed and /or down-regulated MCF7 cells. The results indicated that CSE reversely regulates STAT3 protein levels.

To further explore the interaction of STAT3 and CSE in breast cancer cells, the reverse regulated effects of CSE on STAT3 expression were investigated. WB showed that CSE overexpression or knockdown distinctly increased or decreased STAT3 and pSTAT3 protein levels in MCF7 cells (Figure 7). The results suggested that CSE could reversely regulate STAT3 expression and consequently enhance the regulated effect of STAT3 on CSE.

## DISCUSSION

In the present study, we discovered that CSE was overexpressed in both human breast cancer tissues and breast cancer cell lines. CSE knockdown suppressed the proliferation and migration of breast cancer cells while CSE overexpression promoted them. These results suggested that CSE might function as a potential tumor promoter in breast cancer. Moreover, we found that STAT3 positively relates to CSE expression and STAT3 could regulate CSE transcription by directly binding to its promoter region.

CSE, one of the endogenous H_2_S synthases, is a pyridoxal-5′-phosphate (PLP)-dependent enzyme that catalyzes the conversion of cystathionine into L-cysteine at the last step in trans-sulfuration pathway and then L-cysteine is further metabolized to yield H_2_S [17]. CSE is prevalently expressed in many tissues, such as liver, kidney, heart, vasculature, ileum, pancreatic islets and placenta [18]. CSE/H_2_S system is implicated in various cellular functions, such as cell growth, differentiation, migration, apoptosis and cell cycle progression [18]. Endogenous H_2_S appears to be involved in many physiological, including vasorelaxation, angiogenesis, cellular energy production, neuromodulator, cytoprotection [19–21] and pathological processes, especially including inflammation and angiogenesis which are closely related to the tumorigenesis [22]. While compared with the bio-functional research of endogenous H_2_S, the investigation about H_2_S-producing enzyme is not enough, especially in the field of tumorigenesis. In this article we focused on the biological functions of CSE (endogenous H_2_S synthase) in breast cancer.

Cell proliferation, cell cycle, apoptosis and migration are associated with tumor development and progression. Here, we observed that CSE may promote human breast cancer cell growth due to the proliferation inhibited by knocking down CSE and facilitated by over-expressing CSE. S phase arrest caused by CSE down-regulation may be the reason why endogenous H_2_S could promote cellular proliferation and cell viability. Apoptosis analysis showed that there was no significant change in CSE knockdown cells comparing with their parent ones. Cell migration inhibited by CSE knockdown and facilitated by CSE over-expression was also observed. The data indicated that CSE might function as a potential tumor promoter.

To clarify the relative level of CSE in tumor, we determined CSE expression in breast cancer tissues and cell line and found CSE was strongly expressed (Figure 1). Accidentally the positive correlation between STAT3 and CSE expression was observed in breast cancer tissues and cell lines (Figure 5A–5F). Moreover, CSE was decreased markedly both at mRNA and protein levels when STAT3 was knockdown in breast cancer cells (Figure 5G–5I). So we hypothesized a novel mechanism, which was the potential role of STAT3 in regulating CSE expression and H_2_S level. STAT3 is a member of the STAT family with important roles in cellular transformation, proliferation, inflammation, and metastasis of cancer [23]. As a transcription factor, STAT3 regulates a wide variety of gene expression and consequently mediates critical cell functions [24–25]. So to investigate whether STAT3 regulates CSE expression at transcription level, we searched the potential STAT3 transcription factor-binding sites in CSE promoter using Jaspar (http://jaspar.genereg.net/) and identified several STAT3 transcription factor-binding sites in CSE promoter region.

To explore the mechanism of CSE gene expression regulated by the STAT3 pathway, the full CSE promoter luciferase plasmid and a series of truncated and deleted CSE promoter luciferase plasmids were constructed. The dual-luciferase reporter assay results showed that the promoter pCSE4 (−504 to −286) presented the strongest activity compared with the other ones, representing the core promoter. The data indicated that CSE is a direct target of STAT3. Moreover, we also found that CSE could reversely regulate STAT3 expression (Figure 7).

**Figure 8:**
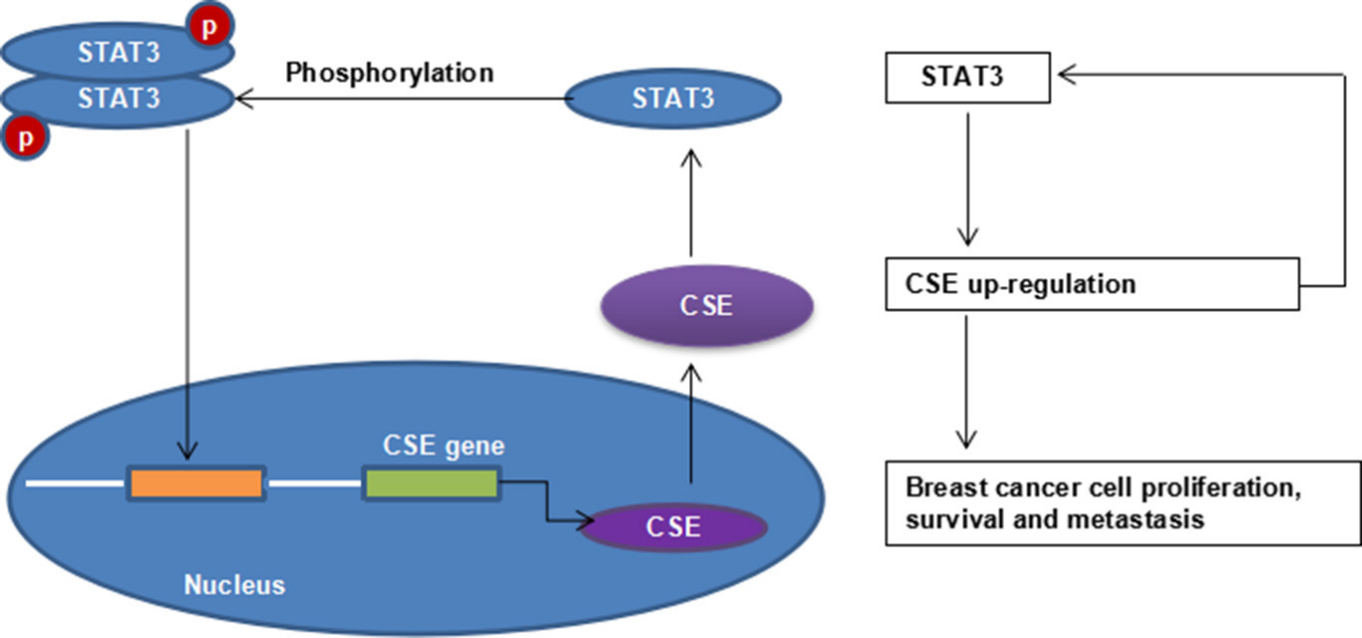
STAT3 and CSE interaction schematic diagram

In summary, we demonstrated for the first time that CSE/H_2_S system promoted breast cancer development and progression in association with the STAT3 signaling pathway (Figure 8). The study provides novel insights on STAT3-regulated CSE expression. Furthermore, these findings highlight CSE/H_2_S system inhibitors as innovative candidates for the treatment of breast cancer.

## MATERIALS AND METHODS

### Patient samples and cell lines

All 60 breast cancer tumor and adjacent non-tumor samples from patients were recruited from the Huaihe Hospital in Kaifeng, China. This study was approved by the ethics committee of Medical School, Henan University. Human breast cancer cell line MCF7 and Mammary epithelial cell lines (Hs578Bst and MCF 10A) were obtained from the American Type Culture Collection (ATCC, Manassas, VA, USA), and cultivated in Dulbecco’s modified Eagle’s medium (DMEM) supplemented with 10% fetal bovine serum (Zeta-life, USA) in a 37°C incubator with 5% CO_2_.

### RNA extraction and qRT-PCR

Total RNAs were extracted using TRIzol Reagent (Invitrogen, Carlsbad, CA, USA), and genomic DNA elimination and complementary DNAs synthesis were performed using PrimeScriptTM RT reagent Kit (RR047A, Takara, Dalian, China). qPCR was performed with SYBR Premix Ex Taq II, and the PikoReal™ Real-Time PCR System (Thermo Fisher Scientific, Inc.) was used to measure messenger RNA (mRNA) expression. The reactions for each sample-primer set were performed in triplicate. Relative quantification analysis was performed using the comparative Cq (2-ΔΔCq) method [16]. All data were normalized to the internal control GAPDH. Primers as followed: CSE: forward: 5ʹ- CCCATCTCACTGTCCACCAC -3ʹ, reverse: 5ʹ- GTGCTGCCACTGCTTTTTCA -3ʹ, Product length: 115bp; STAT3: forward: 5ʹ- CTGTGGGAAGA ATCACGCCT -3ʹ, reverse: 5ʹ- ACATCCTGAAGGTG CTGCTC -3ʹ, Product length: 128bp; GAPDH: forward: 5ʹ- CTCTGCTCCTCCTGTTCGAC-3ʹ, reverse: 5ʹ- ACCAAATCCGTTGACTCCGA-3ʹ, Product length: 109 bp.

### Immunohistochemistry and WB analysis

Immunohistochemical staining of surgical specimens from breast cancer patients was performed in serial sections of formalin-fixed, paraffin-embedded tissues. After deparaffinization, slides were placed in 0.01M citrate salt solution (Epitope Retrieval Solution) and heated in a microwave oven for 7 min. After cooling and washing with PBS, endogenous peroxidase was blocked by 30 % H_2_O_2_ for 10 min and incubated with 5% BSA to block nonspecific binding of antibodies. The slides were then incubated with CSE primary antibody (1:100; Proteintech Group, Inc., Chicago, IL, USA) at 4°C overnight, followed by biotin conjugated secondary antibody and streptavidin horseradish peroxidase (HRP) for 10 min respectively. Antigen-antibody complexes were visualized in DAB (3,3′-diaminobenzidine), cells were stained with hematoxylin and dehydrated, and then photographed. All incubation steps were done at room temperature.

40 µg of protein was separated by 10% SDS-PAGE and transferred to polyvinylidene difluoride membrane (Millipore Corporation, Bedford, MA, USA) at 70 mA for 2 h. The membrane was then blocked in 5% fat-free milk, and probed with specific primary antibodies against STAT3 and CSE at 4°C overnight. After incubation with the secondary antibody, the proteins were visualized using an EasyBlot Enhanced Chemiluminescence kit (Sangon Biotech Co., Ltd., Shanghai, China) and detected using a FluorChem Q Multifluor System (ProteinSimple, San Jose, CA, USA). GAPDH was used as a loading control. Primary antibodies were CSE rabbit polyclonal antibody (1:1000; Abcam, Cambridge, MA, USA), STAT3 rabbit monoclonal antibody (1:1000; Cell Signaling Technology, Danvers, MA, USA), and GAPDH mouse monoclonal antibody (1:1000, Biyuntian, China). Secondary antibodies were horseradish peroxidase-conjugated goat anti-rabbit and horseradish peroxidase-conjugated goat anti-mouse (1:10,000, Proteintech Group, Inc., Chicago, IL, USA).

### siRNA and plasmid transfection

For knockdown, MCF7 cells in 6-well plates were transfected with Scramble siRNA (Sc siRNA) or specific siRNA against human CSE (Invitrogen, Shanghai, China) or specific siRNA against human STAT3 (Invitrogen, Shanghai, China) using Lipofectamine 2000. The medium was replaced at 6 h post-transfection, and silencing efficiency was determined by WB 48 h after transfection. CSE-specific siRNA sequence (sense: 5ʹ-GGUUUAGCAGCCACUGUAAdTdT-3ʹ; antisense: 5ʹ-UUACAGUGGC UGCUAAACCdTdT-3ʹ); STAT3-specific siRNA sequence (sense: 5ʹ-CCCGUCAACAAAUUAA GAAdTdT-3ʹ, antisense: 5ʹ-UUCUUAAUUU GUUGACGGGdTdT -3ʹ).

pCMV-EGFP vector and pCMV-EGFP-hCSE were purchased from Genechem Co. (Shanghai, China). pCMV-FLAG vector and pCMV-FLAG-hSTAT3 were given as a present by Military Medical Sciences. The plasmids were transiently transfected using Lipofectamine2000 Reagent (Invitrogen) according to the manufacturer’s instructions. Six hours later, the cells were exposed to fresh medium. The CSE stably transfected MCF7 cells were screened under G418 (Sigma). Cell clones were obtained by the cylinder method.

### Quantification of H_2_S concentration

H_2_S determination was performed using the methylene blue method. Briefly, MCF7 and MDA-MB-231 cells, via transfecting with CSE siRNA or pCMV-EGFP-hCSE or exposing to DL -propargylglycine (PAG, Sigma Aldrich, Saint Louis, MO, USA), were treated with 2 mM L-cysteine and 0.5 mM pyridoxal phosphate. Meanwhile, 1% (w/v) zinc acetate (500 µl) was added to the filter papers adhered to tissue culture plate cover to absorb H_2_S. After 48 h, the filter papers were put in the tubes containing 0.2% (w/v) N, N-dimethyl-p -phenylenediamine -dihydrochloride dye (500 µl), 10% (w/v) ammonium ferric sulfate (50 µl) and 3 ml H_2_O and incubated for 20 min at room temperature. Absorbance at 670 nm was subsequently monitored. Production of H_2_S was determined using a standard curve of NaHS (0–1 mM; R ^2^ = 0.9997) and presented as nmol·min ^−1^ per 1 × 10 ^6^ cells.

### Cell viability, proliferation, migration and apoptosis assays

Cells were classified into CSE knockdown group and CSE overexpression group. In the CSE knockdown group, cells were pretreated with CSE siRNA or 2 mM PAG for 48 h. In the CSE overexpression group, cells were pretreated with CSE over-expressed plasmid for 48 h. Each sample was tested with at least three replications. Cell viability was performed via MTS assay. Cell proliferation was detected with EdU assay which was performed by plating cells into 96-well dish and staining the cells according to the protocol of the EdU assay kit. The scratch wound assay was used to determine the cell migration. Cells were seeded into 6-well plate and scraped with 10 μl pipette tip at approximately 90% confluency to generate scratch wound and rinsed twice with PBS. Then cells were cultivated in the medium with 5% FBS and the distance was measured at the beginning and after 12 h, 24 h and 48 h. Meanwhile, cell cycle and cell apoptosis were investigated with flow cytometry.

### Construction of CSE promoter reporter plasmid

The human CSE full promoter (−1475/ +197) and five different regions (−1475 to −876, −900 to −724, −748 to −487, -504 to -286,-310 to +197) of the CSE promoter were constructed by PCR amplification and inserted into the pGL3 basic vector using SacI and XhoI restriction enzyme sites. The deletions of binding sites (CTGATGAGAA) were introduced into the promoter plasmid.

### Dual luciferase assay

The 293T cells were plated in 24-well plates (4 × 10^4^ cells per well) in triplicate for each condition. After overnight incubation, cells were transfected with a DNA mix containing pGL3-CSE promoter-luciferase or pGL3-CSE promoter-luciferase1-5, pCMV-FLAG-STAT3 or empty vector, and pRL-TK plasmids. Luciferase activities were measured at post-transfection 48 h using a Dual-luciferase reporter kit (Vigorous, Beijing, China). Each experiment was repeated three times.

### Statistical analysis

Statistical analyses were performed with the SPSS 17.0 software (SPSS, Inc., Chicago, IL, USA). Data are expressed as mean ± s.d. Differences between two groups were analyzed using one-way ANOVA, followed by Student’s *t*-tests. *p* < 0.05 was considered statistically significant.
